# Predictive value of the single most suspicious ultrasound feature in subcentimeter thyroid nodules: a retrospective observational cohort study

**DOI:** 10.1007/s00432-024-05895-z

**Published:** 2024-08-06

**Authors:** Peiying Huang, Lili Han, Xiulin Shi, Fangsen Xiao, Qingbao Shen, Xuejun Li, Fuxing Zhang

**Affiliations:** 1 Department of Endocrinology and Diabetes, The First Affiliated Hospital of Xiamen University, School of Medicine, Xiamen University, Xiamen, China; 2 Xiamen Diabetes Institute, Xiamen, China; 3 The School of Clinical Medicine, Fujian Medical University, Fuzhou, China; 4 Department of Endocrinology, Fuzhou First General Hospital Affiliated with Fujian Medical University, Fuzhou, Fujian China; 5 Department of General Surgery, The First Affiliated Hospital of Xiamen University, School of Medicine, Xiamen University, Xiamen, China

**Keywords:** Thyroid nodule, Ultrasound, Thyroid cancer

## Abstract

**Purpose:**

Proper management of subcentimeter thyroid nodules remains challenging for both clinicians and patients. Conducting extensive sonographic research using a safe and inexpensive tool for identifying thyroid nodules is necessary. The aim of this study was to identify whether having the highest-risk ultrasound (US) characteristic suggests that US-guided fine-needle aspiration (FNA) biopsy of subcentimeter nodules is more appropriate for the identification of malignancy than active surveillance (AS) or surgery.

**Methods:**

The data of patients with highly suspicious subcentimeter thyroid nodules and US characteristic data who underwent surgery were retrospectively examined.

**Results:**

Among a total of 556 subcentimeter nodules, 223 (40.1%) were benign, and 333 (59.9%) were malignant, with a mean maximal nodule size of 8.1 mm. In addition to age younger than 45 years, several US features were significantly associated with malignancy: irregular margins, the presence of microcalcifications, and taller-than-wide shapes (*P* < 0.001). Multivariate analysis also revealed that a taller-than-wide shape (OR = 8.988, *P* = 0.0015) was an independent factor associated with malignancy in subcentimeter thyroid nodules. The diagnostic performance of preoperative FNA was classified as a malignancy, with a sensitivity of 98.4%, specificity of 100%, positive predictive value of 100%, and negative predictive value of 76.9%.

**Conclusions:**

This is one of the few reports based on actual data of the most suspicious US features in subcentimeter thyroid nodules. A taller-than-wide shape US feature is most significantly associated with malignancy. FNA is a simple, accurate, and reliable preoperative method for diagnosing malignant subcentimeter thyroid nodules with highly suspicious US characteristics. AS was less appropriate than FNA for subcentimeter nodules with a taller-than-wide shape, especially in patients ≤ 45 years of age.

## Introduction

The widespread use of ultrasound (US) screenings and US-guided fine-needle aspiration (FNA) biopsies has led to a rapid increase in the number of identified thyroid malignancies in recent decades. This in turn has led to an increasing trend of overdiagnosis and overtreatment of small papillary thyroid carcinoma (PTC) (Vaccarella et al. 2015; Furuya-Kanamori et al. 2016). The revised American Thyroid Association (ATA) guidelines, published in 2015 (Haugen et al. 2015), recommend FNA biopsy only for nodules > 1 cm in size with US feature patterns that appear intermediately and are highly suspicious. However, FNA biopsy can also be used to assess nodules in the subcentimeter range that exhibit highly suspicious US features. Furthermore, a large percentage of thyroid nodules that do not meet the FNA indications are aspirated in medical practice (Jeong et al. 2020), reflecting risk aversion among clinicians who are concerned about missing malignancies.

Active surveillance (AS) is a possible alternative to surgical treatment for subcentimeter PTC, especially when cytopathological findings (refer to the Bethesda System) (Ali et al. 2023) are convincing and there is no evidence of local invasion, metastasis, or aggressive disease (Haugen et al. 2015). As the rates of disease-specific mortality and disease recurrence are minimal and thyroid surgery for the treatment of subcentimeter PTC has no proven benefits, the ATA (2015), ACR (2017) (Tessler et al. 2017), and European Thyroid Association (2017) guidelines (Russ et al. 2017) (Table 1) recommend the management of subcentimeter thyroid nodules with AS. However, while AS can be used to avoid the complications and adverse events associated with surgery, patients who are concerned about tumours remaining untreated or metastasizing are at risk of experiencing psychological stress (Yeh et al. 2015), especially when US reveals thyroid nodules that are highly suspicious of malignancy. Therefore, as reported in a previous study, the number of FNA biopsies or surgeries has increased rather than decreased because of the frequent detection of nodules in the subcentimeter range that appear highly suspicious on US. Moreover, patients with malignant subcentimeter nodules generally opt for surgery rather than AS (Jeong et al. 2020).

**Table 1:** Comparison of the ATA, ACR-TIRADS and EU-TIRADS high malignancy risk stratification and FNA decision-making guidelines

Scoring system	Sonographic pattern	US feature	Malignancy risk, %	FNA decision
ATA	High suspicion	Solid hypoechoic nodule or solid hypoechoic component of a partially cystic nodule with one or more of the following features: irregular margins (infiltrative, microlobulated), microcalcifications, taller-than-wide shape, rim calcifications with small extrusive soft tissue component, evidence of ETE	> 70 to 90	FNA at ≥ 1 cm
ACR-TIRADS	TR5: highly suspicious	≥ 7 points (select one feature from each of the categories: composition, echogenicity, shape, margin and echogenic foci, then sum the points)	> 20	FNA if ≥ 1 cm Follow if ≥ 0.5 cm
EU-TIRADS	EU-TIRADS 5: high risk	At least 1 of the following highly suspicious features: Irregular shape Irregular margins Microcalcifications Marked hypoechogenicity (and solid)	26–87	< 10 mm FNA or active surveillance, FNA if > 10 mm

The revised ATA guidelines define highly suspicious US features as a solid or mainly solid hypoechoic nodule with at least one of the following characteristics: microcalcifications, taller-than-wide dimensions, rim calcifications with a noticeable soft-tissue component, irregular margins, or extrathyroidal extension (ETE) (Haugen et al. 2015). The American College of Radiology Thyroid Imaging Reporting and Data System (ACR TI-RADS) assigns three points to microcalcifications or taller-than-wide dimensions (Tessler et al. 2017). Thus, both guidelines define high-risk US characteristics of malignancy similarly.

In this study, we investigated whether the presence of relatively high-risk US characteristics indicates that subcentimeter nodule FNA is more appropriate for the identification of malignancy than AS or surgery to help clinicians evaluate benign or malignant subcentimeter nodules during follow-up.

## Materials and methods

### Study design and patient population

We retrospectively assessed the data of patients with thyroid nodules in the subcentimeter range exhibiting highly suspicious characteristics on US who had undergone surgery at our hospital from July 2018 to December 2019. We applied the following inclusion criteria: (1) preoperative US showing features highly suggestive of malignancy, (2) surgical treatment, and (3) diagnosis via pathological examination after surgery. The exclusion criteria were as follows: (1) incomplete preoperative US data, (2) thyroid nodules not confirmed via pathological examination, and (3) previous thyroid surgery.

### Assessment

Two physicians experienced in thyroid US performed the examinations. US nodule features, clinical characteristics, US features (nodule size, content, echogenicity, margins, calcification, shape, and presence of cervical lymph nodes), and histopathology results after surgery were recorded. Patients with solitary thyroid lesions showing no involvement of the nodes underwent hemithyroidectomy, and those with multifocality, ETE, or lateral lymph node metastasis were treated with total thyroidectomy. Routine central neck dissection (bilateral or ipsilateral) was performed prophylactically during partial and total thyroidectomy, and the levels of free triiodothyronine, free thyroxine, thyroid-stimulating hormone, thyroid peroxidase antibodies, and thyroglobulin were assessed before surgery.

**Fig. 1:**
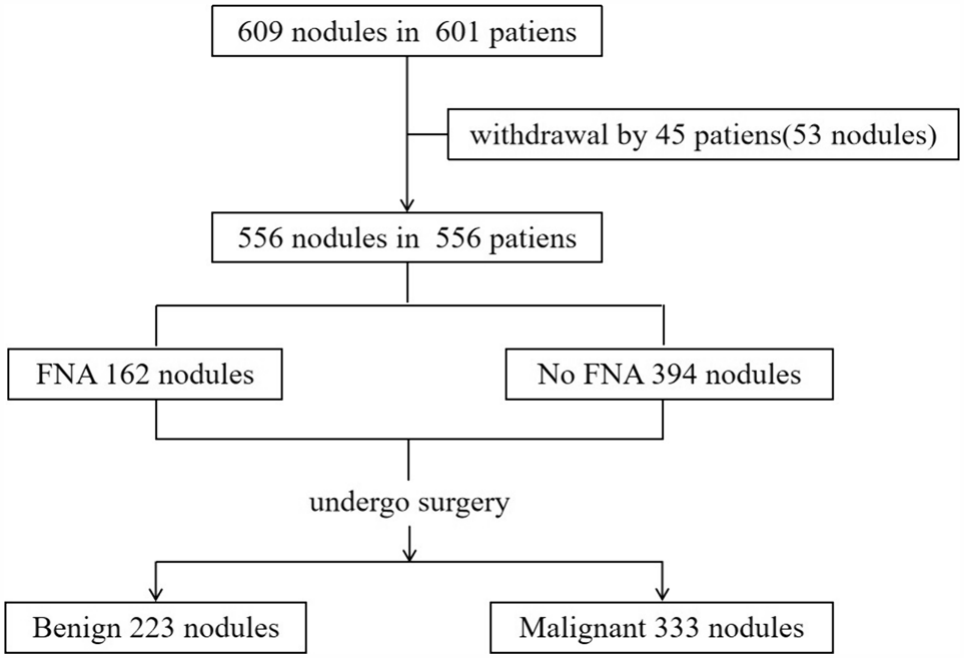
Flow chart of the study

In this study, a total of 609 subcentimeter thyroid nodules in 601 patients were assessed, and the remaining 556 nodules in 556 patients had US features that were classified as highly suspicious; 223 nodules (40.1%) were benign, and 333 (59.9%) were malignant (Fig. 1).

### Ethical approval

The Institutional Review Board of the First Affiliated Hospital of Xiamen University approved the study protocol (2020-No.035).

### Statistical analysis

Categorical variables were analysed using Chi-square tests or Fisher's exact test, while rank-sum tests or independent t tests were applied to continuous variables. Clinical factors and US features with significant differences were evaluated using multivariate logistic regression analysis to identify characteristics that are independently associated with malignant thyroid nodules and their invasive biological behaviours (cervical lymph node metastasis).

We applied the following criteria to the cytopathology results of subcentimeter nodules that had undergone preoperative FNA to determine the sensitivity, specificity, positive and negative predictive value (PPV, NPV), and diagnostic accuracy. Nodules were determined to be FNA-positive when they had been given the label “suspicious for malignancy” or “malignancy” (both corresponding to a highly suspicious ultrasonographic pattern according to the ATA guidelines); those labelled “benign” were considered FNA-negative. Statistical analyses were conducted using SAS version 9.3, and a *P* value < 0.05 was considered to indicate statistical significance. The 95% confidence intervals are displayed for all the results.

## Results

In the present study, the mean maximal nodule size was 8.1 mm. Table 2 lists the detailed characteristics of all the nodules. The following malignancies were diagnosed: papillary carcinoma (n = 331), medullary carcinoma (n = 1), and follicular carcinoma (n = 1). Univariate analysis revealed that irregular margins, microcalcifications, taller-than-wide dimensions, and age < 45 years were factors associated with malignancy (*P* < 0.001) (Table 1), and rim calcification with small extrusive soft tissue components and extrathyroidal extensions were not observed in the subcentimeter nodules included in our study. Furthermore, there was no difference in sex between patients with benign and malignant thyroid nodules. Multivariate analysis revealed that taller-than-wide dimensions (OR = 8.988,* P* = 0.0015) were independently associated with malignancy in thyroid nodules in the subcentimeter range (Table 3).

**Table 2:** Characteristics of 556 subcentimetre thyroid nodules with highly suspicious US features

Characteristics	Malignant (n = 333)	Benign (n = 223)	P value
Sex			0.3703
Male	53 (15.92)	42 (18.83)	
Female	280 (84.08)	181 (81.17)	
Age (years)			0.0038
< 45	191 (57.36)	100 (44.84)	
≥ 45	142 (42.64)	123 (55.16)	
US features			
Margin			0.0002
Circumscribed	209 (62.76)	155 (69.51)	
Irregular	124 (37.24)	68 (30.49)	
Calcification			< 0.0001
Without microcalcification	189 (56.76)	183 (82.06)	
With microcalcification	144 (43.34)	40 (17.94)	
Shape			< 0.0001
Wider than tall	191 (57.36)	202 (90.58)	
Taller than wide	142 (42.64)	21 (9.42)	

Numbers in parentheses are percentages

*US* ultrasound

**Table 3:** Multivariate analysis of predictors of malignancy in subcentimetre thyroid nodules

Characteristics	Odds ratio	P value
Age younger than 45 years	1.868 (0.808, 4.316)	0.1438
Irregular margin	1.214 (0.220, 6.694)	0.8235
Presence of microcalcification	1.180 (0.425, 3.279)	0.7509
Taller-than-wide shape	8.988 (2.322, 34.785)	0.0015

Numbers in parentheses are 95% confidence intervals

The 333 subcentimeter malignant nodules were classified according to US characteristics and clinical features—lymph node metastasis was identified in 57 nodules (17.1%). According to the univariate analysis, taller-than-wide dimensions on US were significantly associated with lymph node metastasis (*P* < 0.01), while age younger than 45 years was also identified as a significant risk factor (*P* < 0.05). However, calcifications, irregular margins, and patient sex did not significantly predict subcentimeter thyroid malignant nodules with lymph node metastasis (Table 4). We found that all 223 histologically benign nodules had the following highly suspicious US features: irregular margins (30.49%, n = 68), microcalcifications (17.94%, n = 40), and taller-than-wide shapes (9.49%, n = 21). FNA cytopathology was performed on 162 of the 556 subcentimeter nodules before surgery (Table 5). The diagnostic performance of the preoperative FNA for correctly classifying a nodule as “malignant” was high, with a sensitivity of 98.4%, a specificity of 100%, a PPV of 100%, and an NPV of 76.9%.

**Table 4:** Characteristics of microcarcinomas with lymph node metastasis according to clinical characteristics and US features

	Lymph node metastasis (n = 57)	P value
*Characteristics*		
*Age (years)*		0.015
< 45	35 (61.40)	
≥ 45	22 (38.60)	
*Sex*		0.391
Female	31 (54.39)	
Male	26 (45.61)	
*US features*		
Margin		0.992
Circumscribed	28 (49.12)	
Irregular	29 (50.88)	
*Calcification*		0.174
Without microcalcification	33 (57.89)	
With microcalcification	24 (42.11)	
*Shape*		0.005
Wider than tall	47 (82.46)	
Taller than wide	10 (17.54)	

Numbers in parentheses are percentages

*US* ultrasound

**Table 5:** Distributions of cytologic diagnoses and cytologic-histologic correlation

Bethesda system^a^ cytological diagnosis	Number of nodules according to cytologic diagnoses	Histopathological diagnoses					Risk of malignancy per diagnosis (%)
		Benign	Malignant				
			PTC	FTC	MTC	ATC	
Unsatisfactory	7 (4.3%)	2	5	0	0	0	71.4
Benign	13 (8.0%)	10	3	0	0	0	23.1
AUS/FLUS	18 (11.1%)	9	9	0	0	0	50.0
FN/SFN	3 (1.9%)	2	1	0	0	0	33.3
SUSP	70 (43.2%)	0	69	0	1	0	100
Malignancy	51 (31.5%)	0	51	0	0	0	100
Total	162	23	139				87.0

*AUS/FLUS* atypia of undetermined significance/follicular lesion of undetermined significance, *FN/SFN* follicular neoplasm/suspicious for follicular neoplasm, *SUSP* suspicious for malignancy, *PTC* papillary thyroid carcinoma, *FTC* follicular thyroid carcinoma, *MTC* medullary thyroid carcinoma, *ATC* undifferentiated thyroid carcinoma

^a^Ali et al. (2023)

## Discussion

Proper management of subcentimeter thyroid nodules remains a great challenge for both clinicians and patients. Extensive research on ultrasonography, which is a safe and inexpensive tool for identifying thyroid nodules, is therefore important. While the 2015 ATA guidelines estimated a 70–90% malignancy risk for thyroid nodules > 1 cm in size that appear highly suspicious on US, data for subcentimeter nodules are still lacking. In our sample, 59.9% of highly suspicious thyroid nodules in the subcentimeter range were classified as malignant on postoperative pathological examination. We found that the characteristic US finding of taller-than-wide dimensions allows for the best prediction of malignancy; similar to the results of Frates et al. (2005) and Lee et al. (2011), when this feature was present, the risk of having a malignant tumour was approximately nine times greater than when it was absent. Other studies have also suggested that a taller-than-wide shape on US predicts malignancy with the highest specificity (Ito et al. 2007; Ahn et al. 2010). Specifically, the anteroposterior diameter of the thyroid cancer nodule is usually larger than the transverse diameter, which may be related to the fact that tumour cells in the anteroposterior direction are in the division phase, while those in other directions are relatively static (Moon et al. 2011).

The sensitivity of microcalcification for subcentimeter malignant nodule detection in our sample was low; a previous study reported a sensitivity of 15.2% (Popowicz et al. 2009). Furthermore, we did not observe rim calcifications with ETE or noticeable soft-tissue components, which indicates that these two US features are rarely expressed in subcentimeter thyroid nodules. Earlier studies have investigated risk factors for poor prognoses in patients with papillary thyroid microcarcinoma (PTMC) and have reported that ETE, capsular invasion, and neck lymph node metastasis are independently associated with distant metastasis and nodal recurrence (Mercante et al. 2009; Pisanu et al. 2015). However, the treating physician does not have easy access to such results when assessing patients for subcentimeter nodules before an intervention, which can lead to a poor prognosis. In addition, PTMC is no longer recognized by the WHO 5th edition as an independent histological subtype. This was also the case in this study, where we observed no capsular invasion or ETE and identified only 3 patients with suspicious neck lymph nodes before preoperative US, but lymph node metastases were observed in 57 of 333 malignant nodules by postoperative pathology confirmation. However, we also found that both the characteristic US findings of a taller-than-wide shape and age younger than 45 years were significant risk factors for subcentimeter malignant thyroid nodules with lymph node metastasis and the invasive biological behaviour of malignant tumours. These results are consistent with those of Baier et al. (2009) and Rago et al. (2010).

The presence of highly suspicious US findings helps determine which thyroid nodules require FNA biopsy and reduces the likelihood of over- or misdiagnosis. According to the usual guidelines, FNA should be performed when nodules are > 1 cm in size and have highly suspicious features. However, for thyroid nodules less than 1 cm, existing guidelines generally recommend AS. However, there is no clear documentation for follow-up management. Thus, there is a gap in the literature regarding the appropriate treatment for subcentimeter nodules. In other words, the available guidelines provide clear recommendations regarding thyroid biopsy, and they do not offer a clear and suitable management approach for patients who do not undergo a biopsy. Some studies found a significantly greater frequency of AS in patients with benign biopsy results (83%) than in those without suspicious nodule cytopathology (36%) (Genere et al. 2020). Interestingly, more than 80% of nodules with benign results were still recommended for continued AS. This may stem from the hesitation of clinicians to dismiss the suspicious appearance of a nodule and diagnose it as cytologically benign due to the high probability of malignancy (Brito et al. 2014). The use of AS also reflects a lack of confidence in being able to rule out malignancies. In addition, patients can feel psychologically burdened by concerns about tumours remaining untreated or metastasizing (Yeh et al. 2015) and might want to take future quality of life outcomes into account when making treatment decisions. This study clearly shows that many patients with subcentimeter thyroid nodules that appear highly suspicious on US choose to undergo surgery in our region as well as in China in general. Before performing FNA in such patients, the clinician needs to determine which nodules are most at risk of malignancy.

The results of this study suggest that subcentimeter thyroid nodules that appear highly suspicious on US, particularly those with taller-than-wide dimensions, the most suspicious ultrasound feature, may be recommended for FNA; however, regarding the choice between AS and surgery, because of this patient population’s greater risk (approximately nine times) of having a malignant tumour, the patient’s preference needs to be taken into account. The diagnostic accuracy of FNA in our sample was greater than expected, with high sensitivity and specificity and a 100% PPV. FNA is therefore a simple, accurate, and reliable preoperative diagnostic method for detecting thyroid nodules in the subcentimeter range that appear highly suspicious on US. It can also help in diagnosis and has proven clinical value. If a patient is younger than 45 years and has a nodule with a diameter < 1 cm and a taller-than-wide shape, AS should not be considered for management. Instead, FNAs or surgical interventions could be selected according to the patient's preference, and patients experiencing fear or anxiety related to the progression of their disease should be offered additional support.

We found that one specific malignant US feature alone cannot be used to determine the malignancy risk of a thyroid nodule; this must be done based on a combination of multiple signs. An increase in the number of suspicious features thus suggests a subsequent increase in the risk of malignancy. Because of the limited number of patients with two or more suspicious features on US, we did not conduct further research, which may have reduced the statistical power for identifying differences. The risk of malignancy increases with the accumulation of highly suspicious US features, reaching 97.9% if all highly suspicious US features are present (Jin Young et al. 2011).

The current study has the following advantages. (1) There was a large sample size. (2) Compared with those in other studies, all subcentimeter nodules with suspicious features within our cohort were diagnosed by pathological examination, with relatively few nodules undergoing puncture cytopathology and postoperative pathology. (3) Our separate analysis of the correlations between single US features and pathological properties of subcentimeter thyroid nodules may provide useful reference data for clinical management.

### Limitations of the study

A limitation of this study is its single-centre retrospective observational cohort design. We excluded patients who had not undergone surgery or who were potentially missing many highly suspicious nodule US features that were not confirmed pathologically but were benign. Additionally, some enrolled patients exhibited two to three highly suspicious US features, thus increasing the likelihood of malignancy. This may have resulted in a bias towards the ‘A’ malignancy rate of 59.9% in this study. Furthermore, we were not able to comprehensively correct for some potentially confounding factors. For example, the patient's family history, occupational factors (i.e., radiation exposure), body mass index, and eating habits (i.e., excessive iodine intake) have been associated with an increased risk of thyroid cancer (Babić Leko et al. 2023). Prospective studies are warranted to further verify the results of this study. Health-related quality-of-life questionnaires, the Thyroid Cancer-Specific Quality of Life questionnaire, and the Fear of Disease Progression Questionnaire may also be useful in determining a patient's status when deciding between surgical and nonsurgical treatment. The presence of features in each of the categories in Table 5 was associated with a greater risk of malignancy (ROM) than expected according to the 2023 Bethesda System for Reporting Thyroid Cytopathology. We selected to puncture subcentimeter nodules with highly suspicious US features, which may have led to a greater proportion of postoperative pathological malignancies. In addition, the limited number of patients who underwent FNA may bias the results, and in our future research, more subcentimeter nodules need to be punctured before surgery.

## Conclusions

This is one of the few reports based on actual data of the most suspicious US features in subcentimeter thyroid nodules. Our multivariate analysis revealed that a taller-than-wide shape is the US feature most significantly associated with malignancy. FNA biopsies and surgical operations are often performed in clinical settings, especially for subcentimeter nodules. Moreover, AS may not be the best choice for subcentimeter nodules with taller-than-wide dimensions on US, especially for patients younger than 45 years of age. This was also identified as a significant risk factor for subcentimeter nodules because of the high malignancy rate and often aggressive behaviour.

## Data Availability

No datasets were generated or analysed during the current study.
